# Case Report: Neonatal Urinary Ascites Without Hydronephrosis: A Rare Case of Anterior Urethral Valve and Diverticulum in Preterm Newborn

**DOI:** 10.3389/fped.2022.920817

**Published:** 2022-06-30

**Authors:** Yue He, Sam Bill Lin, WenXuan Li, YuBo Sun, LiangFeng Tang, Rong Zhang

**Affiliations:** ^1^Department of Neonatology, Children’s Hospital of Fudan University, Shanghai, China; ^2^Department of Urology, Children’s Hospital of Fudan University, Shanghai, China

**Keywords:** anterior urethral valve, anterior urethral diverticulum, neonate, urinary ascites, case report

## Abstract

The deformation of congenital obstruction of the anterior urethra is rare in male infants. The anterior urethral valve (AUV) and diverticulum are not common causes of distal urethral obstruction, which may be significant but difficult to diagnose in time. We describe a premature infant who was diagnosed with AUV as part of a diverticulum in the anterior urethra and was presented as massive urinary ascites without hydroureters and hydronephrosis. After indwelling abdominal tube and urinary catheterization, the infant’s massive ascites were resolved, while urethral obstruction had successful treatment by Holmium laser. We suggest that the presence of urinary ascites in fetuses and neonates should be considered as a warning against urinary malformations.

## Introduction

Anterior urethral valve (AUV) is a rare congenital obstructive disorder in males. It is formed from semilunar folds, arising from the anterior urethral floor. The location of AUV can be varied from the external orifice of the urethra to the membranous urethra (1). Anterior urethral diverticulum (AUD) is defined as the anterior urethra protruding outward through the corpus spongiosum with lower urinary tract symptoms, including dysuria, penile swelling, urethral obstruction, and urinary retention (2). Although much rarer, approximately one-third of patients with AUV can be combined with AUD (3).

Due to a variety of urinary obstruction spectrums, the symptoms of AUV could be sometimes very subtle, and clinical presentation ranged from early prenatal diagnoses to severe cases to delayed diagnoses (4). Among neonates, who were diagnosed with AUV prenatally, the most common prenatal ultrasonographic findings were bilateral hydronephrosis and distended bladder (5). However, we diagnosed a preterm infant with AUV whose prenatal and postnatal ultrasound showed massive urinary ascites without obvious signs of urinary system obstruction. In this report, we describe the rare clinical manifestations, diagnosis, and treatment of AUV and diverticulum. Furthermore, we discuss our experience with occult and atypical AUV in neonates.

## Case Presentation

A late preterm male with a birth weight of 2,710 g, born *via* cesarean section because of prenatally diagnosed fetal abdominal ascites, fetal heart rate instability, and polyhydramnios to a gravida 2 para 2 mother at 34 2/7 weeks of gestational age. The mother underwent limited prenatal care, but prenatal ultrasounds are done at 31 weeks, and 33 weeks revealed normal kidneys, but massive fetal ascites without apparent causes.

**FIGURE 1:**
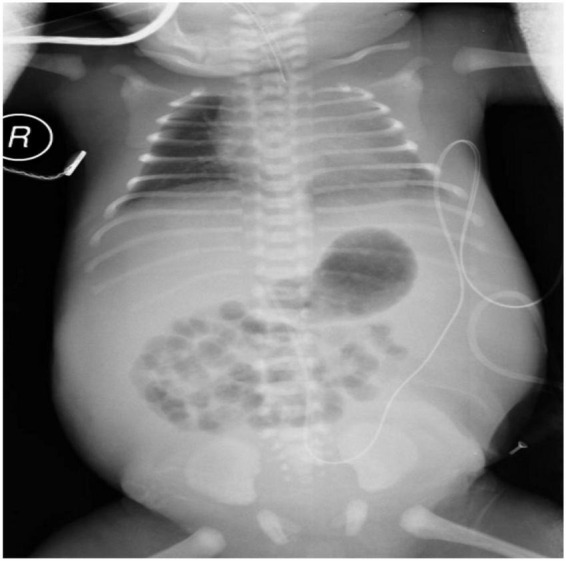
Radiograph of the neonate’s chest and abdomen showing generalized edema and tense distended abdomen with a mount of ascites.

At delivery, the infant was extremely edematous with a seriously distended abdomen. The Apgar score was 1 at 1 min (Apgar scores at 5 min and 10 min were not recorded). Intubation and abdominocentesis were performed at once. After initial stabilization, at 4 h after birth, he was transferred to our neonatal intensive care unit (NICU) for further evaluation and treatment. Physical examination during admission found skin edema, tense distended abdomen with an indwelling abdominal catheter, and tachypnea on mechanical ventilation (Figure 1).

**FIGURE 2:**
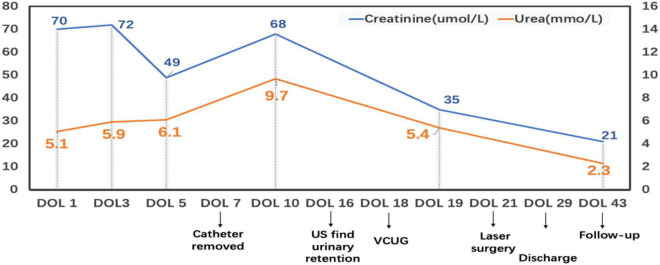
This broken line graph shows the variation of the blood urea and creatinine during the hospital and follow-up. DOL: Day of life; US: ultrasound; VCUG: Voiding cystourethrogram.

The ultrasound showed an indistinct structure of bilateral kidneys, without hydronephrosis or dilatation of the ureters. An abdominal CT scan showed massive ascites. A small patent ductus arteriosus was detected on Echocardiogram. The initial postnatal radiograph did not show any evidence of pleural or pericardial effusion. The Coombs’ test and toxoplasmosis, rubella, cytomegalovirus, herpes simplex, and HIV (TORCH) screening were negative. The blood type was A positive Rh-positive. No evidence of portal vein hypertension or congenital heart disease was found. Blood chemistry taken in the first few days revealed hypoalbuminemia and creatinine of 70 umol/L (Figure 2). Full blood cells, coagulation function, thyroid function, and other laboratory tests that could cause hydrops were within normal limits. Initial analysis of the ascites fluid showed cell count 15*10^6^/L, total protein 19.2 g/dL, glucose 4.1 mmol/L, creatinine 150 umol/L, and urea nitrogen 7.7 mmol/L. The fluid culture was negative. Based on the elevated creatinine, the urinary ascites were initially suspected, and a 6-F urinary catheter was placed smoothly by the pediatrician 12 h after birth. After being inserted urinary catheter, the infant achieved a urine output of 38 ml immediately.

**FIGURE 3:**
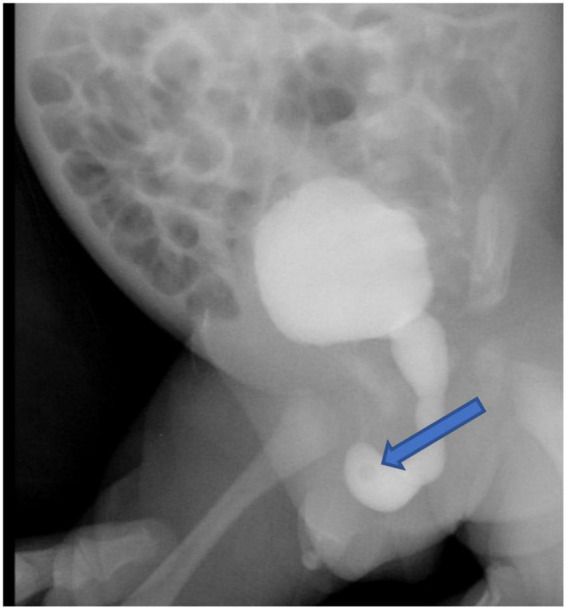
The voiding cystourethrogram showed the obstruction and the proximal dilation of the urethra without vesicoureteral reflux, and a large amount of residual contrast solution in the bladder after voiding. The arrow points to the circular defect of the anterior urethra.

**FIGURE 4:**
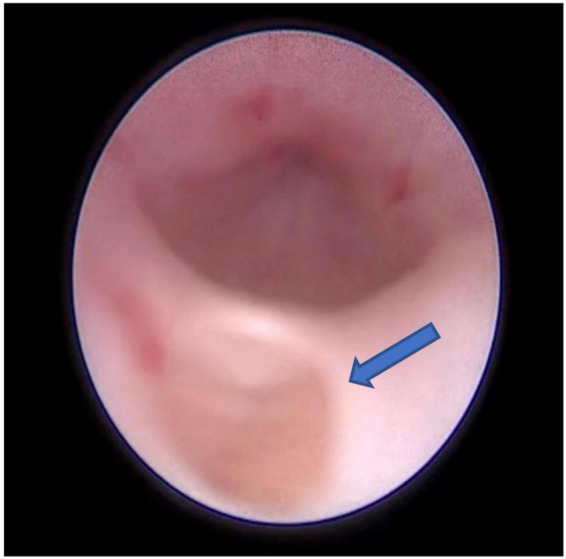
The endoscopic view showing a cusp-like valve as the distal structure of the diverticulum in the anterior urethra, the arrow points to the diverticulum in the 6 o’clock position of the urethra.

The infant had an indwelling abdominal tube for draining ascites, with average drainage of 90 ml daily. Abdominal distention was gradually reduced and ascites subsided smoothly. On day 4 after birth, the infant was extubated for non-invasive ventilation, and enteral feeding was successfully established. On day 7 after birth, abdominal and urinary catheters were removed. There were no accumulations of the ascites with normal daily urine volume. The cause of urinary ascites is difficult to determine accurately. Repeated ultrasound, on day 16 after birth, revealed significant urinary retention without hydronephrosis. Voiding cystourethrogram (VCUG) was performed on day 18 after birth on the urologist’s recommendation, showing a small circular defect of the anterior urethra and obstructive lesion of the anterior urethra (Figure 3). The cystourethroscopy showed a mucosal fold in the urethra, located in the ventral penobulbar urethra along with a diverticulum proximal to the valve, dilation of the proximal urethra, and bladder trabeculations. Therefore, the anterior urethral valve and diverticulum were identified, which had successful treatment by Holmium laser under cystourethroscopy on day 21 after birth (Figure 4). The urethra recovered from obstruction after simple ablation of the mucosal fold in the distal end of the diverticulum. After the procedure, the baby had a normal urinary stream after 7 days of catheterization. He was discharged on day 29 after birth. This confirmed that the infant’s massive ascites and hydrops were caused by the anterior urethral valve and diverticulum.

Finally, the infant is being followed up by the nephrology and urology teams. Urine output and kidney function remained normal. The renal dynamic imaging was done 1 month after discharge which showed fair perfusion of both kidneys and clear excretion. The infant was also followed up at 8 months of age, his parents said that their baby stayed with satisfied voiding, and the urinary tract ultrasound showed normal.

## Discussion

Hydrops fetalis is classified into immune hydrops fetalis (IHF) and non-immune hydrops fetalis (NIHF). About 0.8-2.3% of the NIHF is caused by malformations of the urinary system (6, 7). Fetal hydrops caused by urinary malformation is mostly manifested in the form of urinary ascites or urinoma. Obstructive uropathy or neurogenic bladder are the common causes of urinary malformation (8). Prenatal ultrasound in our case showed a large number of ascites. AUV and diverticulum were revealed postnatally after resolving massive urinary ascites.

The Anterior urethral valve is an uncommon congenital malformation of the lower urinary tract, with an incidence rate of 1/30∼1/7 of the posterior urethral valve (PUV) (1.6∼2.1/10,000) (9). Many cases are not diagnosed until childhood or adolescence, during which time the symptoms persist, leading to irreversible kidney damage (10). The etiology of AUV is still controversial. Some have been proposed as possible mechanisms that are related to embryonic urethral development defects and the valve formed by excessive urethral tissue during embryonic development (11). It has also been reported that AUV and diverticulum formation may result from the distal tip of a ruptured syringocele (12, 13). AUV is manifested as a mucosal fold in the urethra, located generally along the ventral surface of the urethra. During voiding, the fold may rise; when its distal tip rests against the roof of the urethra, urine flow is impeded. When urinating, the diverticulum tends to fill with urine, which causes the distal tip to move forward and upward, resulting in obstruction of the urethra lumen (14).

During the neonatal period and infancy, AUV can cause bladder rupture, urinary ascites, and azotemia, due to severe urinary tract obstruction (15). There are several hypotheses to explain why hydronephrosis did not occur in our baby. The onset time of our infant is during the late pregnancy. However, the AUV is in the ventral bulbar urethra, but bladder condition and appearance were not significantly affected (Figure 3). If the AUV was a congenital urethral defect, it would not remain normal in the third trimester. Congenital structural problems were often progressive which could cause significant problems for the bladder, ureter, and even the kidneys. Thus, we assumed that prenatal AUV formation was caused by the rupture of Cowper’s syringocele. A transitory and sudden increase in urinary pressure resulting from obstruction may lead to rupture of the bladder or extravasation of urine and the development of urinary ascites. Vriees et al. (16) proposed that in terms of long-term results of bladder and renal function, urinary ascites caused by obstructive urinary tract lesions are superior to those without ascites. The relatively high osmotic pressure of the urine can make the hypotonic fluid in the blood vessels or tissue spaces enter the peritoneum, leading to the progressive increase of urinary ascites (17, 18). However, we presume that the bladder rupture of our infant had gradually healed when the urinary catheterization was placed. After the removal of the catheter, the manifestation turned out to be urinary retention rather than urinary ascites under the condition of urinary obstruction.

Gopalkrishna et al. (19) reported a 9-year-old child with AUD, who presented dribbling after micturition, and penile swelling since birth. He was also found to have patent ductus arteriosus and polydactyly. Boissonnat et al. (20) reported a child diagnosed with the prune-belly syndrome with AUD. However, most of the AUV and diverticulum infants were rarely associated with other malformations (21), and that is consistent with our infant, whose genetic test found no related pathogenic variants. Clinical manifestations of AUV infants vary widely, depending on the patient’s age and degree of obstruction, but mostly exhibit urination dysfunction. It has been reported that a third of cases were diagnosed in the neonatal period (21). The predominant symptoms are recurrent fever, urinary tract infections, and pyuria. However, the clinical manifestation of our case is massive urinary ascites, without the symptoms of urinary tract obstruction, such as fever, penile balloon changes, abnormal renal function, ureteral dilatation, and hydronephrosis during prenatal and hospitalization. When the catheter was removed, urine volume and ascites were not affected, making it difficult for the clinician to make a correct diagnosis. If the follow-up urinary ultrasound had not been performed, we could not find the urine retention, and naturally, the AUV and diverticulum may not have been diagnosed. This presents a diagnostic challenge that urethral valve disease may have occult onset and delayed presentation.

The diagnosis rests essentially on voiding cystourethrography, but AUV, sometimes, was insufficient *via* radiograph for the diagnosis. The voiding cystourethrography of our case only showed anterior urethral obstructive disease, while the endoscopic examination could detect the anterior urethral valve and diverticulum. Thus, urethroscopy is more informative in making the correct diagnosis. After surgery, the urine volume and renal function of the infant were normal, and the infant was discharged.

## Conclusion

No cases of neonatal urinary ascites caused by non-obstructive AUV have been reported in previous literature. We suggest that even in the absence of elevated creatinine, abnormal urine volume, or hydronephrosis, the presence of urinary ascites in fetuses and neonates should be considered as a warning against urinary malformations. Clinicians need to be aware of and maintain a high degree of suspicion of AUV. Moreover, prompt diagnosis is essential to avoid delayed treatment and irreversible kidney damage.

## Data Availability Statement

The original contributions presented in this study are included in the article/supplementary material, further inquiries can be directed to the corresponding author/s.

## Ethics Statement

The studies involving human participants were reviewed and approved by the Ethics Committee of the Children’s Hospital of Fudan University. Written informed consent to participate in this study was provided by the patient legal guardian.

## Author Contributions

YH, RZ, and LT contributed to the conception and design of the study. YH and WL organized the database. YH and YS performed the provision of study materials. YH wrote the first draft of the manuscript. SL wrote sections of the manuscript. All authors contributed to the manuscript revision, read, and approved the submitted version.

## Conflict of Interest

The authors declare that the research was conducted in the absence of any commercial or financial relationships that could be construed as a potential conflict of interest.

## Publisher’s Note

All claims expressed in this article are solely those of the authors and do not necessarily represent those of their affiliated organizations, or those of the publisher, the editors and the reviewers. Any product that may be evaluated in this article, or claim that may be made by its manufacturer, is not guaranteed or endorsed by the publisher.
